# Does the use of nest materials in a ground-nesting bird result from a compromise between the risk of egg overheating and camouflage?

**DOI:** 10.1242/bio.042648

**Published:** 2019-12-09

**Authors:** Jesús Gómez, Gustavo Liñán-Cembrano, Cristina Ramo, Macarena Castro, Alejandro Pérez-Hurtado, Juan A. Amat

**Affiliations:** 1 Departamento de Ecología de Humedales, Estación Biológica de Doñana (EBD-CSIC), Calle Américo Vespucio 26, 41092 Sevilla, Spain; 2 Instituto de Microelectrónica de Sevilla (IMSE-CNM), Consejo Superior de Investigaciones Científicas (CSIC) and Universidad de Sevilla, Calle Américo Vespucio 28, 41092 Sevilla, Spain; 3 Instituto Universitario de Investigación Marina (INMAR), Universidad de Cádiz, Campus de Excelencia Internacional del Mar (CEIMAR). Campus Universitario de Puerto Real, 11510 Puerto Real (Cádiz), Spain

**Keywords:** Background matching, Coloration, Disruptive camouflage, Rates of egg heating, Thermal ecology, Trade-off

## Abstract

Many studies addressing the use of nest materials by animals have focused on only one factor to explain its function. However, the consideration of more than one factor could explain the apparently maladaptive choice of nest materials that make nests conspicuous to predators. We experimentally tested whether there is a trade-off in the use of nest materials between the risks of egg predation versus protection from overheating. We studied the ground-nesting Kentish plover, *Charadrius alexandrinus*, in southern Spain. We added materials differing in thermal properties and coloration to the nests, thus affecting rates of egg heating, nest temperature and camouflage. Before these manipulations, adults selected materials that were lighter than the microhabitat, probably to buffer the risk of egg overheating. However, the adults did not keep the lightest experimental materials, probably because they reduced camouflage, and this could make the nests even more easily detectable to predators. In all nests, adults removed most of the experimental materials independently of their properties, so that egg camouflage returned to the original situation within a week of the experimental treatments. Although the thermal environment may affect the choice of nest materials by plovers, ambient temperatures were not so high at our study site as to determine the acceptance of the lightest experimental materials.

## Abstract (precis)

**Summary:** Our study shows that when the thermal environment is not too stressful, plovers choose nest materials to improve egg camouflage, rather than to buffer the risk of egg overheating.

## INTRODUCTION

Many animals increase the survival prospects of their offspring by providing different forms of care, one of which is nest building (Smiseth et al., 2012), which is widespread across animal taxa (Hansell, 2005). Insects, birds and mammals use different materials in the construction of nests, which vary from simple structures to more elaborate ones (Smiseth et al., 2012). Diverse functions have been attributed to bird nests, including structural support for eggs or offspring, concealment from predators, protection from parasites, buffering against environmental hazards, or serving as a signal of the adult’s phenotype (Collias and Collias, 1984; Hansell, 2000; Fast et al., 2010; Mainwaring et al., 2014).

There is much variation of bird nest designs, which not only depend on habitat type and nest location (e.g. tree, ground, cavity, etc.), but also on the multiple roles that nest materials play (Collias and Collias, 1984; Hansell, 2000). Although the materials used to construct nests determine nesting success, there are few experimental studies testing their functions (Deeming and Mainwaring, 2015). In more elaborate nests, there may be several layers, with the outer materials mainly providing structural support, and the lining materials serving mainly for insulation (Hansell, 2000; Biddle et al., 2017). The nests of many ground-nesting birds are very simple, consisting of shallow scrapes into which the birds usually add materials such as pebbles, pieces of vegetation or shell fragments. These materials are collected from around the nests, and this may result in scarcity when bird aggregations are large, eventually leading to the pilfering of nest materials among conspecifics (Carrascal et al., 1995).

The use of nest materials is one of the various strategies used by animals to reduce the hazards of extreme thermal conditions for eggs, and may be especially important for species that breed at ground level in unsheltered sites (i.e. uncovered by vegetation or rocks) that may therefore be exposed to cold winds and/or direct solar radiation (Howey et al., 1984; Hilton et al., 2004; Gedeon et al., 2010; Heenan, 2013; de Zwaan and Martin, 2018). The protection of eggs against extreme thermal hazards may be achieved by different mechanisms (Howell, 1979; Amat et al., 2017; Gómez et al., 2018b), including the use of materials that reduce heat loss by eggs under cold conditions (Reid et al., 2002; Tulp et al., 2012) or heat gain under hot conditions (Mayer et al., 2009). In addition, the materials in ground nests prevent the eggs from coming into contact with water after storms, improving nesting success (Moreno et al., 1995). Finally, the insulator properties of nest materials reduce the energy costs of incubation by adults, leading to a shorter incubation period, which in turn contributes to maximizing egg hatchability (Lombardo et al., 1995; Hansell, 2000; Deeming, 2002; de Zwaan and Martin, 2018).

Materials may also facilitate nesting success in ground-nesting birds by improving camouflage. Indeed, egg background-matching reduces predation risk (Lee et al., 2010; Skrade and Dinsmore, 2013; Troscianko et al., 2016b). The importance of the materials in improving camouflage may be especially significant when the match between the nesting habitats and the eggs is poor. In such cases, the birds may improve egg camouflage with the materials that they add to nests (Amat et al., 2012; Troscianko et al., 2016a). This in turn shows that some birds are selective in their choice of nesting materials (Kull, 1977; Solís and de Lope, 1995; Gómez et al., 2018a).

Because of the importance of egg camouflage for nesting success, it may seem surprising that the materials used in some nests may render them conspicuous to predators (Evans, 1954; Montevecchi, 1976). This indicates that there may be trade-offs among factors that affect nesting success on the choice of nest materials (Mayer et al., 2009; Stoddard et al., 2011). In fact, birds may face a trade-off between egg camouflage and egg temperature in some situations (Hilton et al., 2004; Eggers et al., 2005; Gómez et al., 2016; de Zwaan and Martin, 2018).

Here, we studied experimentally whether there is a trade-off in nest material choices by a ground-nester, the Kentish plover *Charadrius alexandrinus*, between the need for egg camouflage and to avoid egg overheating. This species nests in shallow scrapes, into which the birds add pebbles, mollusc shells and/or plant fragments collected from around the nests (Wiersma et al., 2019). Kentish plovers may experience heavy heat loads during incubation because they breed in sites exposed to direct solar radiation (Grant, 1982; Amat and Masero, 2004, 2009). Sustained egg temperatures above 40°C threaten embryonic survival (Webb, 1987) and unattended Kentish plover eggs may reach such temperatures in a few minutes (Amat and Masero, 2007; Amat et al., 2017). On the other hand, clutch losses due to predation may be as high as 70% in some sites (Lessells, 1984; Fraga and Amat, 1996). Therefore, in this species there may be selection pressures to have well-camouflaged eggs, and to add nest materials that buffer against high temperatures harmful to embryos. However, these pressures may be in conflict, as the best materials for thermoregulation may not necessarily be best for egg camouflage (Mayer et al., 2009). In our experiments, we used materials with different thermal conductance and different effects on egg camouflage. We predicted that if camouflage was the main selective agent on the choice of nest materials, the experimental materials that better matched the eggs and the surroundings (hereafter named the microhabitat) should be retained in higher quantities than those that produced worse crypsis, regardless of their thermal properties. Alternatively, if overheating was the main selective agent, adults should retain more of those experimental materials on which egg overheating was lower, even if such materials meant that the eggs were poorly camouflaged.

## RESULTS

### Camouflage experiment

**Fig. 1.**
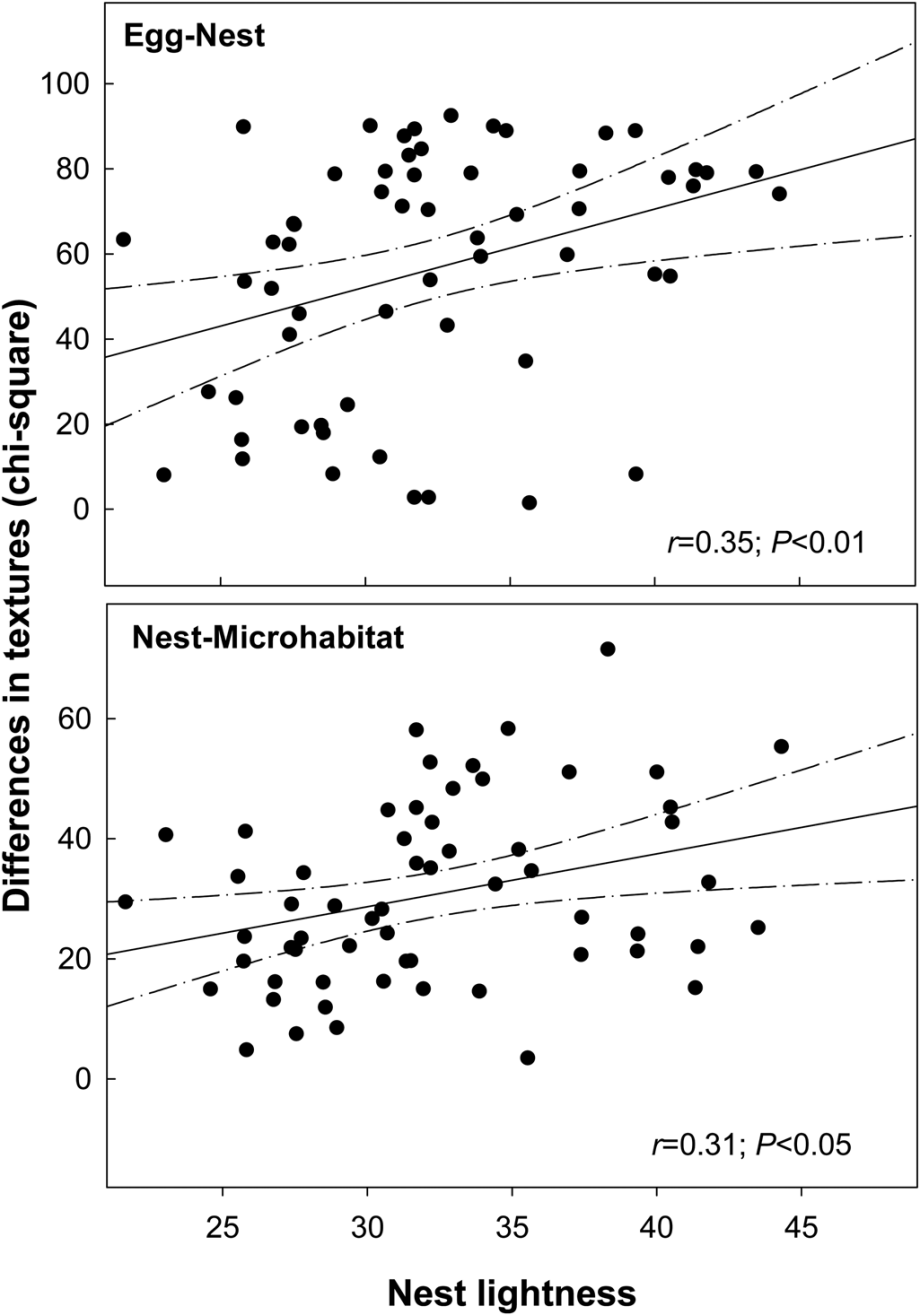
**Relationships between the lightness of nests and background/pattern camouflage.** Slope and associated confidence intervals of the linear regression between lightness of the nests (lab color space) and camouflage of eggs and nests obtained with an analysis of textures. The lower the values of the lightness of nests, the better the camouflage of the eggs with respect to the nests, and that of the nests with respect to the microhabitat.

Under original conditions, before manipulations, the materials selected by nesting birds were lighter (mean±s.e., *L*_nest_=32.28±0.70) than the microhabitats (*L*_microhabitat_=28.42±0.50) and eggs (*L*_egg_=25.31±0.65; *F*_2,118_=56.14, *P*<0.001). The eggs were better camouflaged with respect to the microhabitats than to the nest materials (Egg–Nest_differences_=56.38±3.67 versus Egg–Microhabitat_differences_=41.16±2.07; *F*_1,59_=20.15, *P*<0.001). We found a clear positive relationship between nest lightness and camouflage of either Egg–Nest (*r*=0.35; *P*=0.006) or Nest–Microhabitat (*r*=0.31; *P*=0.014), the greater the lightness of the nest, the worse the camouflage (Fig. 1).

**Fig. 2.**
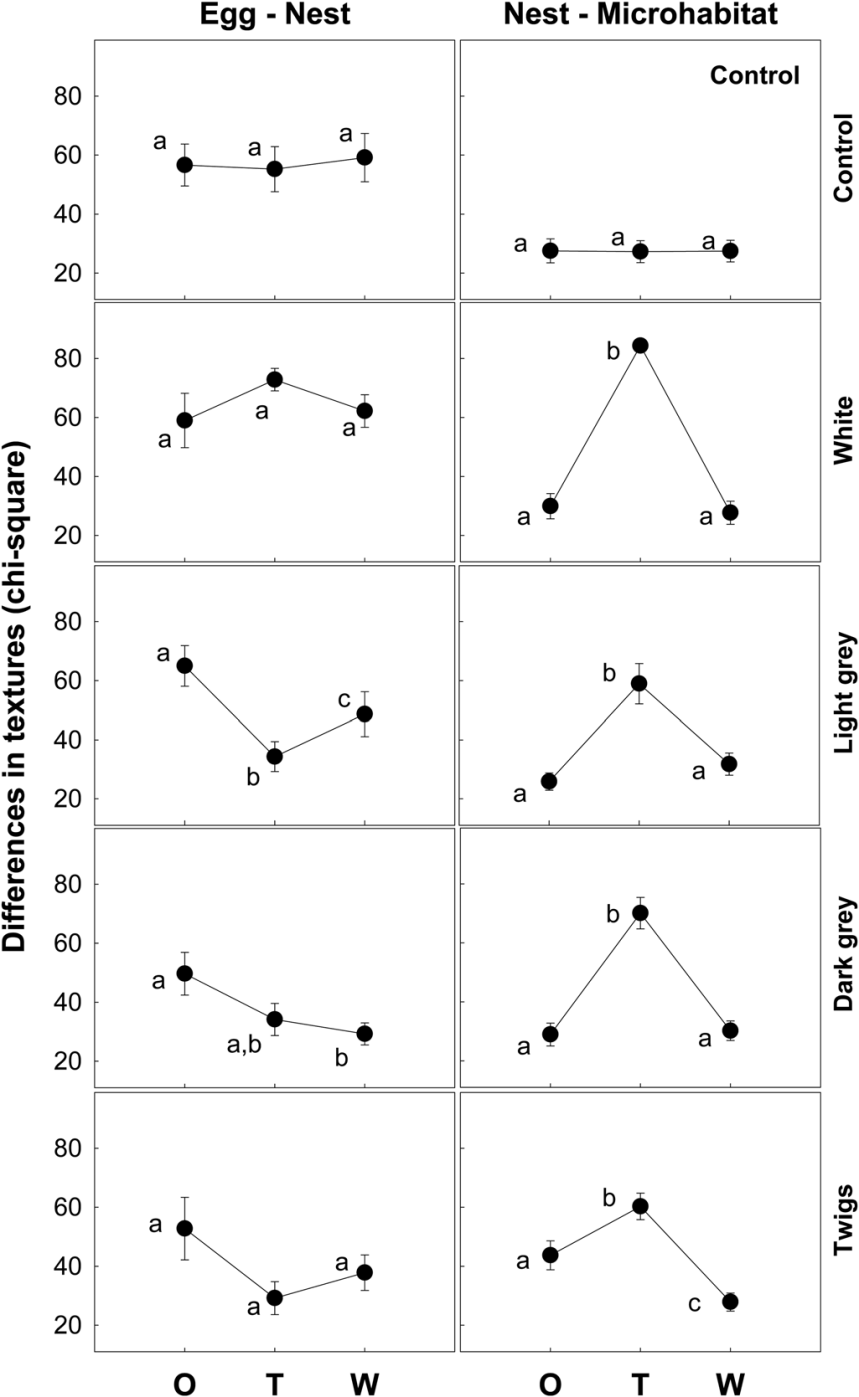
**Variations in background/pattern camouflage during the experiment.** Dots and bars represent, respectively, means and standard errors of camouflage calculated with an analysis of textures in the original situation (O), immediately after the change of nest materials (T), and 1 week after the change (W). The lower the values, the better the camouflage. Different letters denote significant differences (Tukey’s post-hoc comparisons).

Once the original nest materials were replaced by the experimental ones, Egg–Nest camouflage was improved by the addition of light gray pebbles (Fig. 2; *F*_2,20_=13.91, *P*<0.001). However, the contrast between the nest and the microhabitat became greater in all treatments, except in control nests, as expected [Fig. 2; *F*(white)_2,20_=119.91, *F*(light gray)_2,20_=17.78, *F*(dark gray)_2,30_=29.91, *F*(twigs)_2,20_=16.65, all *P*<0.001]. One week after the application of treatments, the adults had removed most of the materials that we had added, leaving only around 15% of the experimental materials. Although the material left when light gray pebbles were added covered a larger area than in the other cases, the differences were not significant (Table 1; *F*_3,43_=1.04, *P*=0.383). The remaining 85% were materials similar to the originals that the adults added to the nest. The removal of the experimental materials by the nesting birds returned camouflage values very close to the original ones. In some cases, the camouflage was even better at the end of the experiment, as occurred with light and dark gray pebbles when considering the camouflage of eggs with respect to nests, as well as with twigs when considering the camouflage of nests with respect to microhabitats. The opposite ­– worse camouflage a week after the addition of experimental materials – never happened.

**Table 1.**
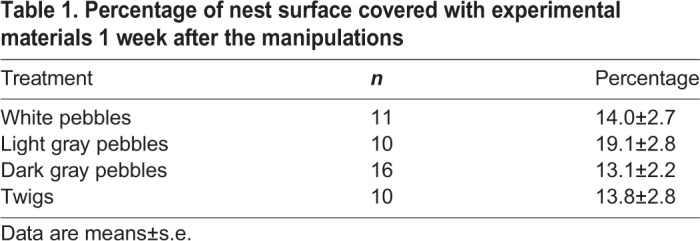
**Percentage of nest surface covered with experimental materials 1 week after the manipulations**

**Fig. 3.**
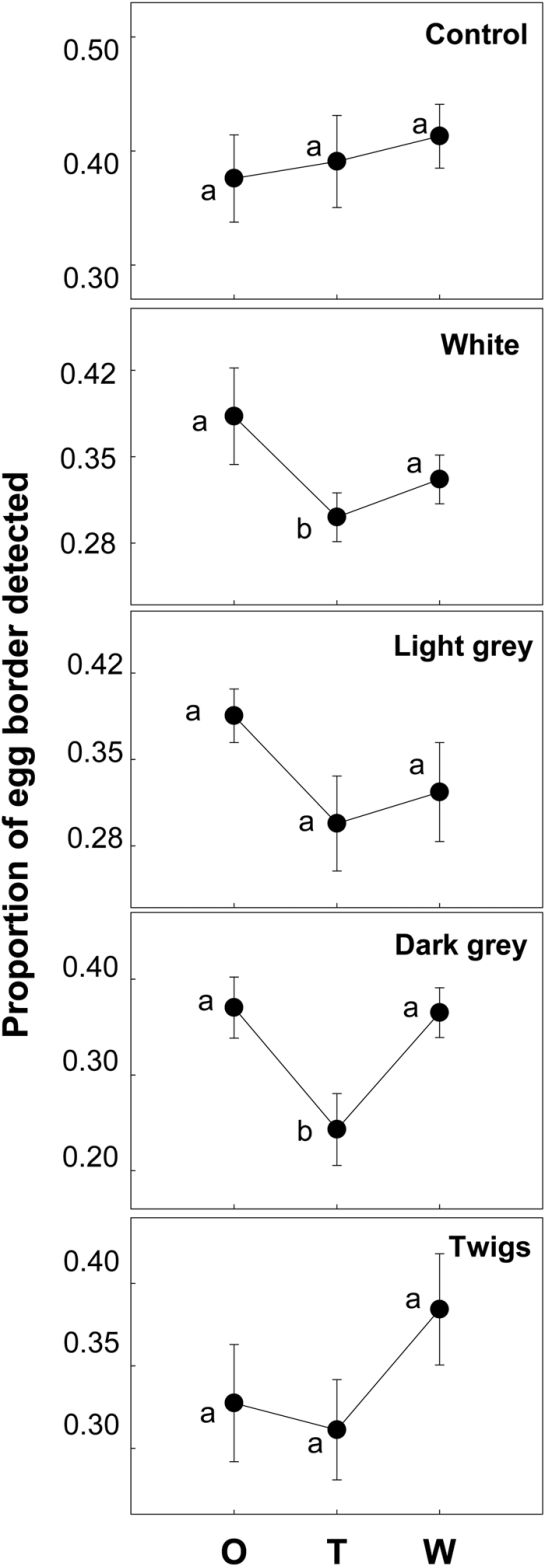
**Variation in disruptive camouflage during the experiment.** Dots and bars represent, respectively, means and standard errors of the proportion of the border of the egg that was detected using a texture analysis in the original situation (O), immediately after the change of nest materials (T), and 1 week after the change (W). The lower the values, the better the camouflage of the eggs. Different letters denote significant differences (Tukey’s post-hoc comparisons).

In terms of disruptive camouflage, only the dark gray and white pebbles produced significant changes, and their inclusion increased egg camouflage [Fig. 3; *F*(dark gray)_2,30_=6.37, *P*=0.005; *F*(white)_2,20_=3.60, *P*=0.046], although after a week the values were similar to the original.

### Heating experiment

When the nest materials were heated under field conditions using solar radiation as the energy source, white pebbles reached significantly lower temperatures than the other materials. By contrast, the dark gray pebbles were the materials that reached the highest temperatures (Table 2, *F*_5,120_=317.33, *P*<0.001). The temperatures reached by the materials were highly and negatively correlated with their total reflectance in the visible range of the light spectrum (Spearman correlation, *r_s_*=−0.83, *P*=0.029). This means that, as expected, the lighter colored materials reached lower temperatures than the darker ones.

**Table 2.**
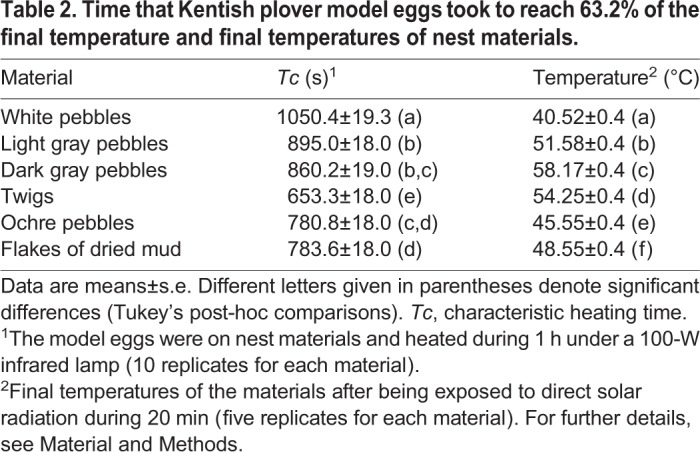
**Time that Kentish plover model eggs took to reach 63.2% of the final temperature and final temperatures of nest materials.**

Similar results were found under laboratory conditions; the eggs on white pebbles had a significantly higher characteristic heating time, *Tc*, than those on the other materials, so they took longer to reach 63.2% of the final temperature (*T_f_*, the temperature reached by experimental eggs after 60 min heating under laboratory conditions) (Table 2, *F*_5,51_=38.38, *P*<0.001). They were followed by eggs on light gray pebbles (Table 2). In contrast, the eggs on twigs had significantly lower *Tc* values than those on the other materials.

## DISCUSSION

According to our predictions, if overheating was the main selective agent on the choice of nest materials, it would be expected that the plovers retain white pebbles, as this is the material that reached the lowest temperature and the eggs on this type of material took the longest time to heat. By contrast, if camouflage was the main selective agent, it would be expected that plovers retain the light gray pebbles, because it is the only material with which the egg–nest camouflage improved. However, our results showed that the Kentish plovers removed around 85% of the experimental nest materials in all cases, and this was independent of how well those materials matched the eggs or their thermal properties. In all treatments, the matching between the nest and the microhabitat significantly worsened, so the decision of the adults to remove most of the experimental nest materials was probably driven by the necessity of increasing the camouflage of the whole system (i.e. Egg–Nest–Microhabitat) and not just some parts of it. We found that in the cases of dark gray pebbles and twigs, materials that reached the highest temperatures, the camouflage (Egg–Nest and Nest–Microhabitat, respectively) improved 1 week after we had applied the treatments, probably because the quantities of such experimental materials left in the nests did not increase the risk of egg overheating, but improved camouflage. However, the opposite never happened, so nest camouflage 1 week after the manipulations was never worse than in the original nests. These results once more support that, of both selective agents (i.e. camouflage and risk of egg overheating), camouflage is the one that was driving the adults' behavior at our study site. Indeed, the camouflage returned to the original values 1 week after we made the changes in the nests, probably because of the importance of egg and nest camouflage for nesting success (Lee et al., 2010; Skrade and Dinsmore, 2013; Troscianko et al., 2016b).

Blue tits, *Cyanistes caeruleus*, reject feathers added experimentally during nest building, and it has been suggested that the risk of cuckoldry could be a reason for the rejection of such feathers, because males would assume that an extra-pair bird was visiting the nest (García-Navas et al., 2013; Mainwaring et al., 2016). However, the risk of cuckoldry would not explain our results, since we supplemented nests during incubation. Indeed, feathers added experimentally during incubation, when there is no risk of cuckoldry, were not removed by blue tits from nests (Mainwaring et al., 2016).

We found that under natural conditions, plovers selected lighter materials for their nests than those available in the microhabitat, despite such materials worsening egg camouflage, which may make the eggs more easily detectable by predators. This may indicate that the thermal properties of nest materials could also affect their choice, suggesting that birds may not just simply select the best microhabitats in terms of thermal properties when the conditions of the environment present extreme temperatures (Carroll et al., 2015), but also that adults may manipulate the thermal environment using nest materials that reduce the risk of egg overheating under hot conditions, even if those materials worsen egg and/or nest camouflage (Mayer et al., 2009).

The environmental temperatures experienced by the plovers during incubation in most of the days at our study site were not very high (see Materials and Methods), and under such conditions the risks of overheating may not be critical for unattended eggs (Fig. S1). It is likely that the occurrence of more extreme environmental temperatures would have changed the decision of the adults to remove the lightest colored experimental nest materials (i.e. those with lower thermal conductance) because the importance of the thermal environment on the trade-off between the risk of egg overheating and camouflage would be stronger in those circumstances (Gómez et al., 2016).

To sum up, camouflage seems to be more important in our study than the risk of egg overheating in determining the choice of nest materials by Kentish plovers, although under natural conditions the adults selected lighter materials that were a worse color match with the eggs, perhaps to prevent egg overheating. The fact that the thermal environment was not likely very extreme at our study site may explain why the adults removed the lightest colored experimental materials, since camouflage may worsen with such materials, otherwise we would have expected that these materials be retained. This may explain why, in some cases, such shorebirds as Eurasian oystercatchers *Haematopus ostralegus* and little ringed plovers *Charadrius dubius*, use very pale nest materials that make their nests conspicuous (Evans, 1954; Montevecchi, 1976; Fig. S2). Finally, and interesting to note, 1 week after the experimental change, the camouflage was better in some cases, which implies that adults may increase the egg/nest camouflage when the availability of different nest materials is higher. This could explain why shorebirds use different materials within the same nest if available, since this heterogeneity of materials may increase nesting success (Colwell et al., 2011), probably as a result of improved matching (via background matching, pattern matching and/or disruptive camouflage).

## MATERIALS AND METHODS

### Study site

Our study was conducted in 2013–2015 and 2017 at a 15-hectare (ha) saltpan in Cádiz Bay Natural Park, southern Spain (36° 30′ 53.4″ N, 6° 09′ 23.3″ W). The saltpan offers a variety of substrates, both natural and others introduced by humans to construct tracks. For this study, we located 61 nests on tracks, of which 42% were on ochre-colored pebbles, 23% on dried mud, 23% on debris of construction material, 7% on light gray pebbles and 5% on dark gray pebbles. All substrates, other than the construction material debris, are of homogeneous color. At our study site, the laying season of Kentish plovers encompasses late February to late June (A.P-H., unpublished). Because one of our aims was to analyze the effect of the thermal environment on the choice of nest materials, we conducted the study during the second half of the nesting season (May onwards), when the highest ambient temperatures were expected. The average maximum air temperature registered at a weather station (Puerto de Santa Maria, 36° 36′ 18ʺ N, 06° 09′ 52ʺ W), located 11 km from our field site, was 21.6°C (17.4–31.7°C) in May and 29.3°C (21.8–41.1°C) in June.

### Camouflage experiment

Each nest was photographed (O, original) as found, i.e. without modifying any aspect of the nest or microhabitat. The original nest materials were then replaced with experimental materials. The original materials were discarded far enough from the nest (>7 m) to ensure that they were not recovered by the plovers. Control nests (*n*=12), were left unmodified. The experimental materials used were dark gray pebbles (*n*=16), white pebbles (*n*=11), light gray pebbles (*n*=11) and twigs (*n*=11), of which the birds did not use twigs and white pebbles because they are not available at the study site.

After we replaced the original materials with the experimental ones, we again photographed each nest (T, treatment). A final photograph was taken a week later (W, week later). Photographs O, T and W were taken from approximately 1 m above the nest to include the microhabitat. In addition, we took another photograph (M, material) closer to the nest (approximately 20 cm) after taking photograph W, with the aim of quantifying the amount of experimental material remaining in the nest. Two of the M photographs (one with light gray pebbles and another one with twigs) could not be analyzed because they were overexposed.

The photographs were taken in RAW format with a Canon EOS-400 camera equipped with Canon EFS 18-55 mm lens. The manipulation of nest materials and the photographic procedure took less than 4 min per nest. Photographs were taken between 09:00 and 11:00 h (GMT) so that lighting conditions were comparable between them.

### Digital image analysis

We adapted a texture analysis (Malik et al., 2001), which encompasses background matching/pattern matching, to measure egg camouflage. The images were processed using custom designed functions for MATLAB ver. R2014a (MathWorks, Natick, MA, USA). In a first step, RAW images were decoded and presented through a Graphical User Interface (GUI) for marking regions of interest (ROIs). We defined four types of ROI for each image; egg area (with all eggs marked), nest area, internal area (a small area around the nest that was not included in the analyses) and, finally, the external area (microhabitat), which covered the rest of the scene in every image (O, T, W). To avoid possible distortion in the outer pixels we eliminated a small region around the border of the picture from the image analysis (Fig. S3). Once all images were marked, we ran a second MATLAB function that evaluated camouflage by partitioning the input image into sets of ‘similar’ pixels following Arbeláez et al., (2011). In this second stage, we first mapped the RAW images to the L*a*b* color space (Commission International d'Eclairage) using the same method for the adapted white point as reported by Amat et al. (2017). This color space has proved useful in studies of camouflage (Nguyen et al., 2007; Lovell et al., 2013; Gómez et al., 2016; Amat et al., 2017). Afterwards, each image channel [lightness L*, red/green value a* (‘redness’), yellow/blue value b* (‘yellowness’)], was passed through a bank of image filters containing 56 members. These members corresponded to 14 different spatial filters, defined at four scales (σ={16, 24, 32, 48} pixels). Each set of filters for a particular scale contained odd-symmetric and even-symmetric Gaussian derivative kernels at five orientations (evenly distributed between 0 and 180°), plus four rotation invariant filters (two Gaussian kernels, two Laplacian of Gaussian kernels). At the end of the filtering process, we obtained 168 (3×56) measurements for each pixel in the original image. These measurements were vectorized and clustered using K-means for 14 cluster centers (Nclusters). Finally, each pixel was assigned to the closest cluster center, and the whole output image just contained integer values in the range (1, Nclusters). Then, we computed the clustering signature of each of the regions defined in the first stage. The clustering signature of a particular region was a vector of 14 components, with each of them representing the relative presence (%) of a particular cluster in this region. The number of cluster centers (14) was defined through a process in which we ran our algorithm for a subset of 10 randomly selected input images and varying Nclusters {6, 8, 10, 12, 14, 16, 20}. We then selected the number of centers minimizing the correlation between the clustering signatures for eggs and external areas. This correlation decreased as the number of cluster centers was increased, and showed a typical saturation curve starting at Nclusters=12. Therefore, we chose Nclusters=14, since the decrease in correlation between eggs and external areas obtained for larger Nclusters was not significant when compared to the extremely high growth of computing time per image.

Together with the clustering signature for each region, we also reported the amount of egg perimeter detected from the clustering analysis, which could be used to quantify disruptive camouflage (Lovell et al., 2013; Troscianko et al., 2017). To do so, we created an image containing only texture boundaries. Afterwards, we computed another image containing the borders of marked eggs, and we dilated (Serra, 1983) each egg's border image using a cross-shaped structuring element with a radius of 1% of the arithmetic mean of the lengths of the eggs in the nest. We then calculated the intersection of the two images, thinned it back to a single pixel width line, and calculated the percentage of egg borders that corresponded to texture boundaries.

The final result from our image processing was a CSV file with one row per input image, where we reported the clustering signatures (14 elements) for the four regions defined in every image together with the amount of egg borders reconstructed from texture frontiers. In addition, for recording purposes, we also saved the resulting texture images (see Fig. S3 for an example of detected textures and egg borders). Normalized texture signatures (%) were considered as a histograms of textures and compared using χ^2^ metrics as in Arbeláez et al. (2011, Eqn 9). Hence, for two normalized signatures, *g* and *h*, their degree of similarity is given by:
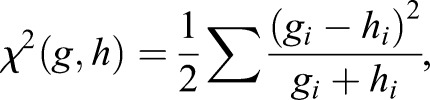
where *i* runs from 1 to the number of textures in the analysis (14). This value can be employed as a measure of camouflage (background matching+pattern matching) with lower values indicating better camouflage.

To quantify how much experimental nest material remained in the nest 1 week after the original materials were removed, we developed a custom tool using MATLAB ver. R2014a (MathWorks, Natick, MA, USA). First, we created a digital grid (squares of 2×2 mm) embedded in the nest ROI of every photo M (Fig. S4). The percentage of squares in which the experimental materials dominated (i.e. covered >50% of the surface of a square) was considered as a measure of the acceptance of those materials by the plovers.

### Heating experiment

To test how the different materials added to the nests produced different thermal conditions for the embryos, we employed a dual experimental procedure. Initially, we measured the temperatures reached by the different materials when directly exposed to the sun using a thermal camera. Additionally, in order to have an indicator of how the heat flowing into the egg varies as a function of the material added to the nest, we measured, under laboratory conditions, the rate-of-change of the temperature inside eggs, filled with Plaster of Paris, which was laid over the different experimental materials.

#### Field conditions

To test how the introduced materials heated up under field conditions, we employed a 640×480 thermal imaging camera (FLIR Systems SC660). The camera was mounted on a tripod 50 cm above ground level and set to take photos every 10 s for 20 min. We placed the materials, receiving direct solar radiation, over a 2 cm thick thermal insulating white polystyrene sheet laid to prevent heat conduction from the ground. Five replicates were used, changing the positions of the materials on the polystyrene sheet to avoid any position-related influence of the thermal conditions. The data were cropped to 1111 s instead of 1200 s (20 min) because some of the final measurements were not recorded in a few replicates.

**Fig. 4.**
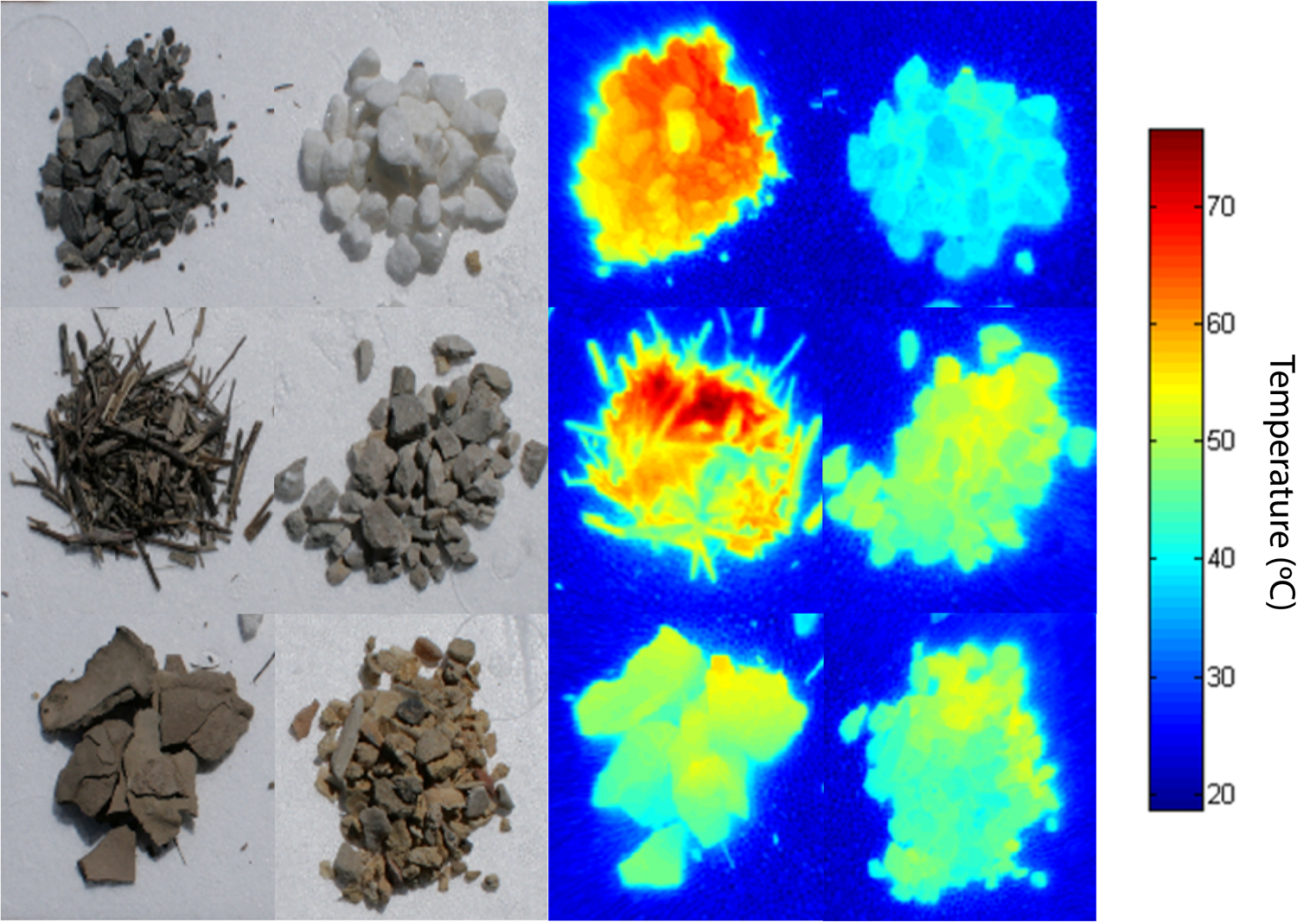
**Visible and thermal images of nest materials.** Left: images of the nest materials acquired with a conventional digital camera. In the left two columns: top left, dark gray pebbles; top right, white pebbles; middle left, twigs; middle right, light gray pebbles; bottom left: flakes of dried mud; bottom right, ochre pebbles. Right: thermal images of the same nests as shown on the left obtained with a FLIRSC660 Thermal Imaging Camera after 20 min of exposure to direct solar radiation.

The raw thermal images (Fig. 4) were decoded using a custom-designed MATLAB function that allowed thermal data to be obtained from the camera output images. Finally, we compiled the results into a CSV file for further analysis. We could not estimate the heating rates of the materials under field conditions because the temperature of the twigs did not fit well to the typical curve for Newton's law of heating, probably because they were more affected by internal air convection than the other materials. For that reason, we just considered the final temperatures reached by each material (averaging the last five records).

We also took a conventional photograph (RAW format) at the beginning of each replicate with a Canon EOS-400 camera equipped with a Canon EFS 18–55 mm lens. We included a gray standard (Lastolite Ezybalance, 30 cm, 18% reflectance) near the nest materials that was later employed for normalization of the images and to obtain reflectance values with the SpotEgg free tool (Gómez and Liñán-Cembrano, 2017).

#### Laboratory conditions

In order to evaluate how the different materials added to the nest created different thermal conditions for the eggs laying on top of such materials, we used a laboratory procedure similar to that of Mayer et al. (2009). Here the goal was to measure the rate of change of the temperature inside Kentish plover eggs (filled with Plaster of Paris) laying over the experimental materials when heated up with an infrared (IR) lamp under controlled laboratory conditions. Under these conditions, by Fourier's law for thermal conduction in an isotropic medium, the rate of change of the temperature inside these filled eggs is proportional to the flow of heat through their surface. Since the only difference between measurements is the material underneath the egg, differences in heat flow (different rate of change of temperatures) will be a function of the material itself and will illustrate how different materials produce different thermal stress conditions.

The nest materials that we used in this experiment were the same as in the field, and we put them in aluminum tins (75 mm diameter, 25 mm depth), and placed a Kentish plover egg filled with plaster of Paris on top (we used four experimental eggs). We also tested the effects of two common materials at the study site on the rate of change of egg temperatures: ochre pebbles and flakes of dried mud, which were not used in the field experiment because of a shortage of available nests. We inserted 30–36 gauge nickel-chromium/nickel-aluminum thermocouple probes (Omega Engineering, Stamford, CT, USA) into the model eggs and these were connected to an Omega OM-550 datalogger, programmed to record every 15 s for 60 min. The egg was heated from 23 cm above using a 100-W IR lamp. Although the thermal conductivity of the Plaster of Paris (0.432 w/mk, Weast, 1986) is very similar to that reported for a natural egg (0.43 w/mk, Henderson, 1963) it is clear that we cannot reproduce the thermal conditions inside a living egg using our experiments. However, in the absence of any thermal regulation activity inside the model egg, and in the controlled conditions of the experiment (same ambient temperature, same energy received from the IR lamp, same humidity) the rate of change of temperature at the center of a ‘model’ egg is directly related to the total heat absorbed, and so, it would confirm the hypothesis that different materials will produce different thermal conditions in a real egg.

**Fig. 5.**
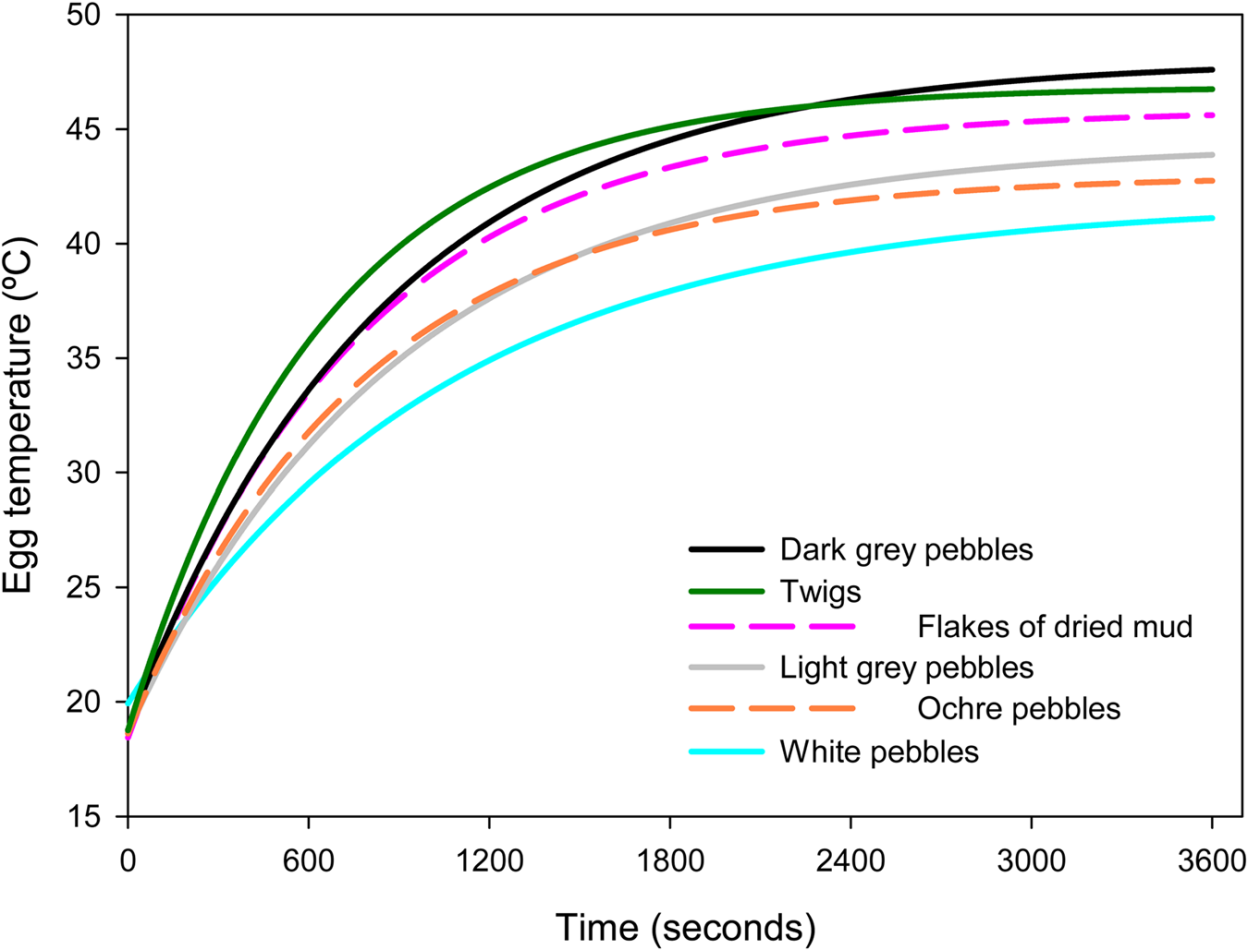
**Temperatures of Kentish plover model eggs placed on different nest materials.** The temperatures were fitted to non-linear models following Newton's law of heating. The model eggs were heated with a 100-W IR lamp for 1 h.

In our operation conditions, heat transfer can be formulated according to Fourier's law, which states that the heat transfer per unit of time per unit area (*A*) (heat flow density) is proportional to the temperature gradient between the objects and negative to its sign;
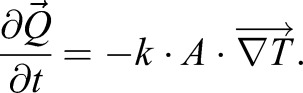


When the temperature gradient within the object under study is negligible (i.e. the object's internal redistribution of thermal energy is much faster than the temperature change rate at its surface) we can assume that the system behaves according to simpler Newtonian cooling (or heating), which states that the rate of temperature change of an object is proportional to the total heat (received or released) *Q*:
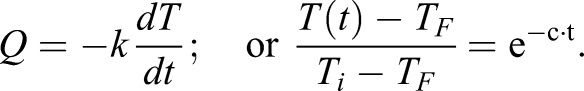


Denoting *c* as (1*/Tc*), leads to:
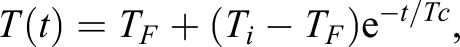


where *T_i_* and *T_F_* are egg temperatures at the beginning and end of the experiment, respectively, *Tc* can be interpreted as the characteristic heating time for each material (i.e. the time at which the total temperature change of the egg on a material is accomplished in 63.2%) and e is the universal mathematical constant which is the base of natural logarithms, this gives a quick insight into how fast an egg heats up under experimental conditions. In order to compare results, we ensured that the heat flow was constant for the different materials in the experiment and that *T_i_* did not vary among treatments (*F*_5,54_=1.63, *P*=0.167, Fig. 5). Under these assumptions, we employed MATLAB Curve Fitting Toolbox (Mathworks, 2018) to fit an exponential model to every different run (egg on a different material type, different physical objects). Thus for every time and temperature series, we calculated a model of the form:
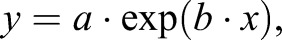


where *y*=*T*(*t*)−*T*_*F*_; *a* = (*Ti−Tf*); *b* =−1/*Tc* and *x*≡*t*.

For every material, ten replicates were fitted, together with their corresponding coefficient of determination (*r*^2^). Finally, model parameters were averaged to obtain mean *Tc* for every material (average *r*^2^±s.e.=0.99743±0.0024; Table 2, Fig. 5).

### Statistical analysis

General linear mixed models with restricted maximum likelihood (GLMM, package ‘nlme’, Pinheiro et al., 2018) were used to test the differences in the final temperatures of the materials. The fixed effect was the type of material with six levels (i.e. the six nest materials), but as we used the five final records to reduce the effect of convection, the random effect was nested: time record/replicate. We used Tukey’s post-hoc tests to assess differences between treatments (‘multcomp’ package, Hothorn et al., 2017). A Spearman correlation was carried out between the total reflectance of the materials and the temperature that they reached under field conditions.

We carried out two GLMMs using the images of the original nests (i.e. before manipulation), one to compare lightness among eggs, nests and microhabitats, and another to compare the degree of camouflage between Egg–Nest and Egg–Microhabitat. Nest identity was the random factor in both models. We also modelled two simple regressions, with Gaussian errors, to analyze the relationship between nest lightness and the degree of camouflage between Egg–Nest and Nest–Microhabitat.

We also used a GLMM to examine variation in camouflage in three occasions: before, immediately after and 1 week after the change of nest materials. We performed three models: Egg–Nest, Nest–Microhabitat and disruptive camouflage for each treatment. The response variable in the first two cases was the differences between textures, whereas the proportion of egg border detected (border ratio) was the dependent variable in disruptive camouflage. The independent variable was the image (O, T and W) and the random factor was nest identity. We used Tukey’s post-hoc test to assess differences between treatments.

Finally, we carried out an ANOVA to test differences among treatments in the quantity of materials that remained in the nest one week after the experimental change.

All statistical analyses were carried out using R statistical software version 3.5.0 (R Core Team, 2018) and the significance level was set at *P*≤0.05.

## Supplementary Material

Supplementary information
