# Supplementary material for: Does the use of nest materials in a ground-nesting bird result from a compromise between the risk of egg overheating and camouflage?
Source: Biol Open. 2019 Dec 9;8(12):bio042648. doi: 10.1242/bio.042648 (PMC6918762; doi:10.1242/bio.042648)
Supplement: Supplementary information [file biolopen-8-042648-s1.pdf]

Fig. S1. Egg and ambient temperatures in the study area. Kentish plover experimental eggs (filled with plaster of Paris and provided with thermocouples) were placed in 4 empty nests and exposed during 15 minutes to direct sunlight at midday. Environmental temperatures (mean ± SE) were measured at exposed sites near nests and 5 cm above ground level (using the same type of thermocouples). Temperatures were recorded every 15 seconds (unpublished data, see Amat et al. 2017 for details). Temperatures above 40ºC (red dashed line) are very critical to embryos (Web, 1987).

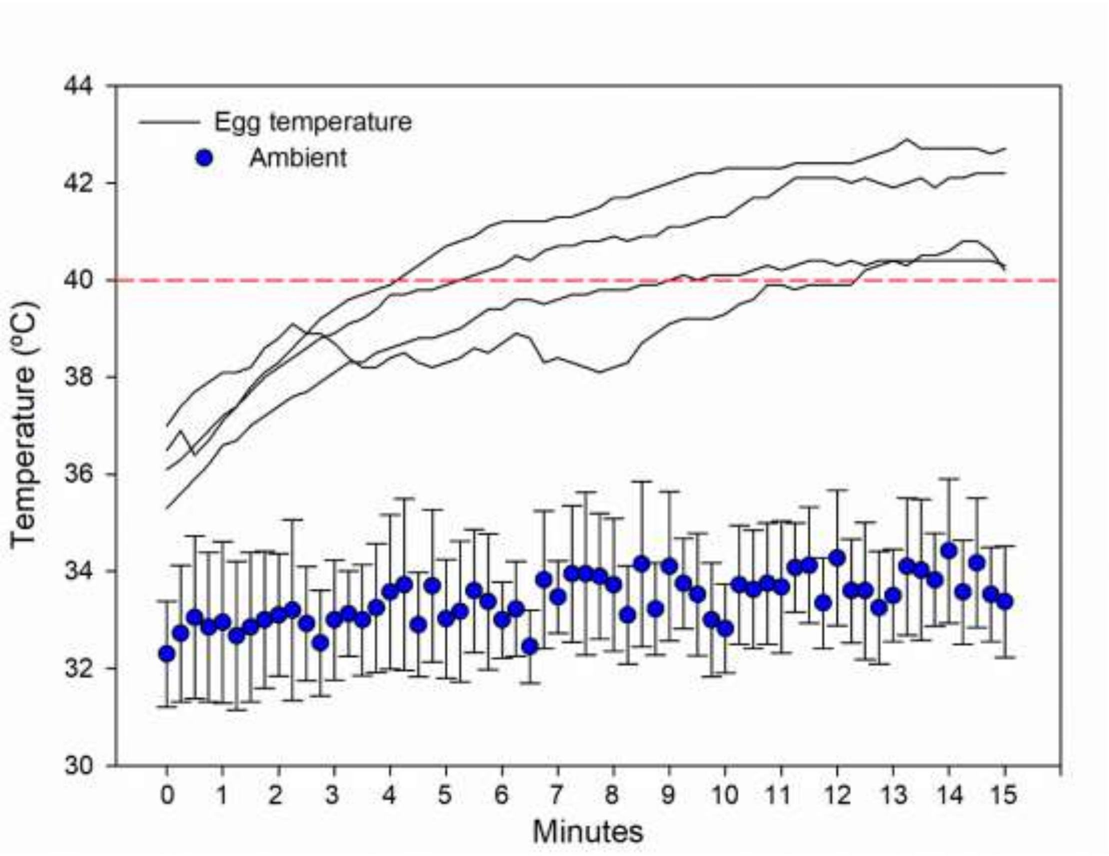

Amat, J. A., Gómez, J., Liñán-Cembrano, G., Rendón, M. A., and Ramo, C. (2017). Incubating terns modify risk-taking according to diurnal variations in egg camouflage and ambient temperature. *Behav. Ecol. Sociobiol.* **71**, 72.

Web, D. R. (1987). Thermal tolerance of avian embryos: a review. *Condor* 89, 874-898.

Biology Open • Supplementary information

Fig. S2. Kentish plover nest. In some cases, the lining may make the nests of Kentish plovers rather conspicuous. This occurs in dark substrates when the birds line the nests with pieces of white shells.

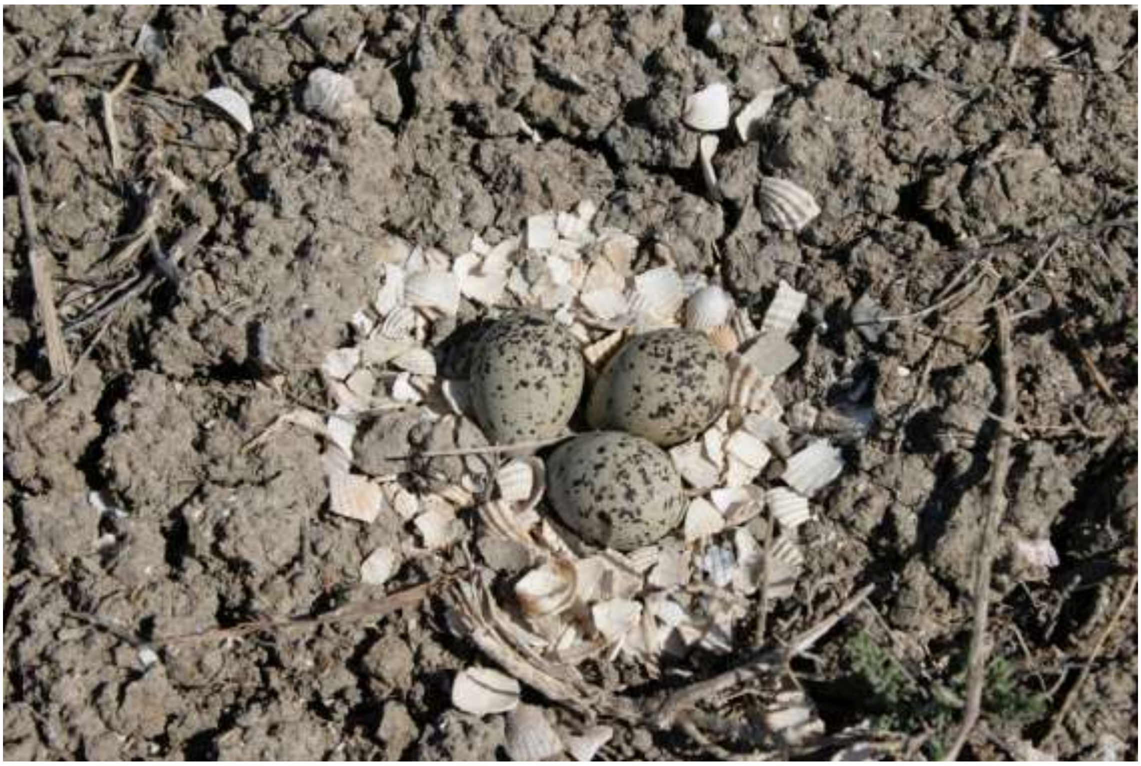

Biology Open • Supplementary information

Fig. S3. Images for texture analysis. A: Original input image with RoIs (selected Regions of Interest). B: Resulting clustering (colours, for representation, are randomly assigned to each cluster [n=14] in each output image) with RoIs. We discarded the image border in the analysis. C: Reconstructed egg borders from texture boundaries (image enlarged).

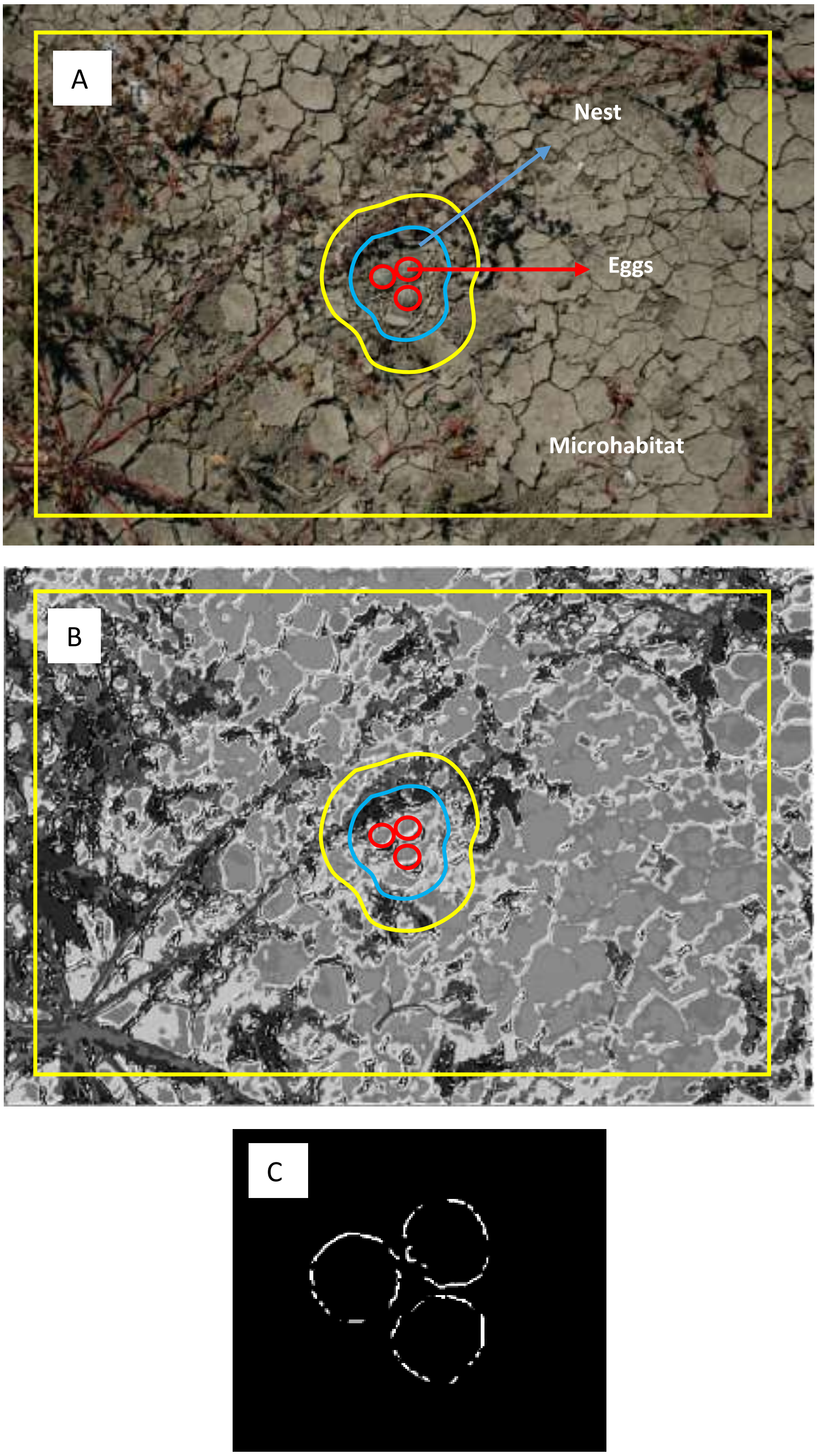

Biology Open • Supplementary information

Fig. S4. Kentish plover nest one week after it was experimentally manipulated. To quantify the remaining experimental materials (in this case dark grey pebbles), we created a digital grit (2×2 mm, in red). The squares with more than 50 % cover with the experimental material were counted as positive (i.e. considered as those in which experimental material was not removed, and are coloured in red here for illustration).

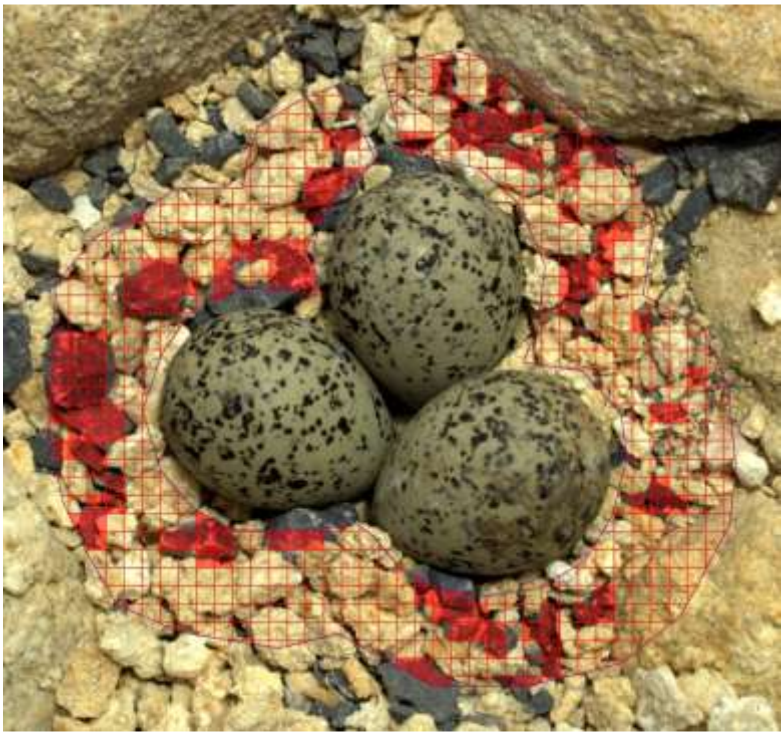

Biology Open • Supplementary information
